# Identification of altered protein abundances in cholesteatoma matrix via mass spectrometry-based proteomic analysis

**DOI:** 10.1186/s40463-015-0104-4

**Published:** 2015-11-25

**Authors:** Derrick R. Randall, Phillip S. Park, Justin K. Chau

**Affiliations:** Section of Otolaryngology – Head & Neck Surgery, Department of Surgery, University of Calgary, Calgary, Foothills Medical Centre, 1403 - 29 Street NW, Calgary, AB T2N 2T9 Canada

## Abstract

**Background:**

Cholesteatoma are cyst-like structures lined with a matrix of differentiated squamous epithelium overlying connective tissue. Although epithelium normally exhibits self-limited growth, cholesteatoma matrix erodes mucosa and bone suggesting changes in matrix protein constituents that permit destructive behaviour. Differential proteomic studies can measure and compare the cholesteatoma proteome to normal tissues, revealing protein alterations that may propagate the destructive process.

**Methods:**

Human cholesteatoma matrix, cholesteatoma-involved ossicles, and normal middle ear mucosa, post-auricular skin, and non-involved ossicles were harvested. These tissues were subjected to multiplex peptide labeling followed by liquid chromatography and tandem mass spectrometry analysis. Relative protein abundances were compared and evaluated for ontologic function and putative involvement in cholesteatoma.

**Results:**

Our methodology detected 10 764 peptides constituting 1662 unique proteins at 95 % confidence or greater. Twenty-nine candidate proteins were identified in soft tissue analysis, with 29 additional proteins showing altered abundances in bone samples. Ontologic functions and known relevance to cholesteatoma are discussed, with several candidates highlighted for their roles in epithelial integrity, evasion of apoptosis, and immunologic function.

**Conclusion:**

This study produced an extensive cholesteatoma proteome and identified 58 proteins with altered abundances contributing to disease pathopathysiology. As well, potential biomarkers of residual disease were highlighted. Further investigation into these proteins may provide useful options for novel therapeutics or monitoring disease status.

## Background

Cholesteatoma is a benign epidermal inclusion cyst that develops within the temporal bone and exhibits locally destructive behavior. Without treatment, cholesteatomas can progressively expand and destroy middle ear and temporal bone structures. This process can lead to secondary infections, which can result in complications such as tympanic membrane perforation, chronic otorrhea, hearing loss, vestibular dysfunction, facial nerve paresis, and intracranial extension [1]. Regardless of surgical technique, cholesteatoma have propensity to recur [2], requiring exteriorization of the middle ear through canal wall down procedures or “second look” tympanomastoidectomy for surveillance of disease in intact canal wall procedures.

Increased understanding of molecular changes in bone through orthopedic and otitis media literature led to theories of cholesteatoma-related bone destruction through cellular resorption, mechanical compression, and second mediator effects [2–7]. Bone erosion involved in the progression of disease may be intrinsic to the cholesteatoma—increased matrix growth factor and cytokine expression, pressure effect of outward growth, and host granulation enzymes. Extrinsic factors include bacterial superinfection, altered osteoclast activity in response to invasion, and changes in bone architecture and cell population [3, 7, 8]. Numerous targeted molecular and genome wide studies on cholesteatoma specimens support some of these hypotheses but fail to appreciate the complex interplay between multiple changes at the cellular level.

Differential proteomic analysis evaluates the active protein constellation between normal and pathologic states [9]. Initiated as two-dimensional gel electrophoresis (2DGE) with densitometry or visual evaluation, modern proteomic analysis evolved to incorporate mass-spectrometry in protein identification from individual 2DGE studies to current chromatographic separation and tandem mass spectrometry techniques capable of analyzing multiple tissue conditions simultaneously. Modern mass spectrometry-based techniques have improved the ability to recognize novel, replicable protein derangements in various diseases and tissues by comparing disease to normal states [10]. These changes can be detected among proteins expressed at very low levels and several orders of magnitude below that of the most abundant proteins.

Previous proteomic approaches to cholesteatoma identified several proteins with altered presence in comparison to post-auricular skin through 2DGE [11]. In this study, we employed a multiplex differential mass spectrometry-based approach termed isotope-tagged relative abundance quantification (iTRAQ) to characterize simultaneously the cholesteatoma matrix proteome in reference to native middle ear mucosa and post-auricular skin as well as to evaluate the bone proteome of ossicles involved by cholesteatoma compared to normal ossicles. With this design we were able to detect agents underlying the pathophysiologic process and destructive behavior demonstrated by cholesteatoma as well as identify and quantify potential biomarkers of disease.

## Methods

### Sample collection

This study received ethical approval from the University of Calgary Conjoint Health Research Ethics Board (REB-14-0883). Patient tissue samples were obtained during primary tympanomastoidectomy from patients with acquired cholesteatoma, whereby cholesteatoma matrix was excised and cleaned of keratin debris; middle ear mucosa was collected from uninvolved regions of the mastoid cavity; post-auricular skin was taken as a 1 mm wide strip of skin along the margin of the skin incision with subcutaneous tissue removed by sharp dissection under an operating microscope. Cholesteatoma-involved ossicles were harvested during tympanomastoidectomy from patients with evidence of ossicular destruction. Control ossicles were obtained from patients undergoing labyrinthectomy for vestibular schwannoma or ossicular reconstruction for traumatic ossicular discontinuity. Tissue samples were immediately placed on ice then stored at −80 °C until sample processing.

### Protein extraction

Samples were thawed on ice then washed three times in 4 °C phosphate-buffered saline containing complete protease inhibitor (Roche, Mississauga, ON). Sample processing was performed by the following methods for either soft tissue or bone, in an identical fashion for each tissue sample, with all buffers containing c0mplete protease inhibitor (Roche, Mississauga, ON).

Soft tissue samples were pooled as mucosa, matrix, or skin samples. Each pool was incubated at 4 °C for 16 h in modified RIPA buffer with protease inhibitor. Samples were then sonicated on ice for 7 s at 20 % power for 5 cycles with 30 s cooling periods. The resultant lysates were centrifuged for 60 min at 12 000 g at 4 °C in an Allegra X-15R centrifuge (Beckman-Coulter, Mississauga, ON) and the supernatant preserved. Equivalent total protein concentrations from each pool were precipitated in 100 % chilled ethanol (Sigma, Oakville, ON) by incubating 24 h at −20 °C followed by 20 min centrifugation at 2500 *g* (4 °C). The pellet was preserved, washed once with 80 % ethanol (v/v), centrifuged at 2500 *g* (4 °C), decanted, and air dried for 5 min. The precipitated samples were stored at −80 °C until iTRAQ analysis.

Individual bone samples were incubated at room temperature in 0.06 M hydrocholoric acid (HCl) for 16 h. The diluent (D1) was removed and the bone samples incubated in 1.2 M HCl at 4 °C for 16 h to decalcify the bone. After decalcification, the diluent (D2) was removed, and bone samples were pooled into two groups (control ossicles or cholesteatoma-involved ossicles) then washed with extraction buffer 1 (E1: 6 M guanidine-HCl and 100 mM Tris–HCl with protease inhibitor). Buffer E1 was added to the pooled samples, which were sonicated on ice for 7 s at 20 % power for 5 cycles with 30 s cooling periods. Samples were incubated for 72 h at 4 °C then centrifuged for 20 min at 12 000 g (4 °C). Supernatant E1 was removed and stored at −80 °C. Samples were washed with extraction buffer 1.5 (E1.5: 6 M guanidine-HCl, 100 mM HEPES, 0.5 M Na_4_EDTA, protease inhibitor), then buffer E1.5 was added to the pellet, agitated, and incubated 72 h at 4 °C. Samples were centrifuged for 20 min at 12 000 g at 4 °C, and supernatant E1.5 removed and stored at −80 °C. Samples were washed with extraction buffer 2 (E2: 6 M HCl, 6 M guanidine-HCl, 100 mM 100 mM Tris, protease inhibitor), then buffer E2 was added to the pellet, fragmented, and incubated 20 h at 4 °C. Samples D2, E1, E1.5, and E2 were combined in their respective pooled samples and precipitated by the same method as the soft tissue samples and stored at −80 °C until iTRAQ analysis.

### iTRAQ reagent preparation

Two LC-MS/MS tracts were defined and run on separate days in order to minimize the impact of high abundance proteins from each tissue type limiting the ability to detect low abundance proteins: one for soft tissue and one for bone samples. Soft tissue and bone lysates were labelled with iTRAQ reagent according to the University of Victoria Protein Centre protocols. 100 μg of total protein from each sample underwent acetone precipitation followed by resuspension in iTRAQ buffer and trypsin digestion. Each protein lysate was labeled with a distinct isotopic iTRAQ reagent: control ossicles (113 Da), cholesteatoma-involved ossicles (115 Da); cholesteatoma matrix 1 (113 Da), cholesteatoma matrix 2 (114 Da), mucosa (115 Da), post-auricular skin (116 Da). These samples were combined and subjected to alkaline (pH 10) reversed phase high performance liquid chromatography on an XBridge C18 BEH300 250 mm X 4.6 mm, 5 μm, 300A HPLC column (Waters, MA, USA), with fractions collected every minute for 96 min.

### LC-MS/MS analysis

Fractions were separated by on-line reversed phase liquid chromatography using a Thermo Scientific EASY-nanoLC II system with a reversed-phase pre-column Magic C-18AQ (100 μm I.D., 2 cm length, and an in-house prepared reversed-phase nano-analytical column packed with Magic C-18AQ (75 μm I.D., 15 cm length, 5 μm, 100 Å; Michrom BioResources Inc, Auburn, CA). The chromatography system was coupled on-line to an LTQ Orbitrap Velos Pro mass spectrometer equipped with a Nanospray Flex source (Thermo Fisher Scientific, Bremen, Germany) and run over a 120 min gradient from 95 % solvent A (2 % Acetonitrile, 0.1 % Formic acid):5 % solvent B (90 % Acetonitrile, 0.1 % Formic acid) to 100 % solvent B. Mass spectrometry data were acquired with a time of flight survey scan of mass range 400–1800 amu where the most abundant ions exceeding 5000 counts and charge state 2–4 selected for fragmentation.

### Data acquisition and analysis

The resulting data were analyzed by Proteome Discoverer 1.4 software suite (Thermo Scientific) using stringency criteria: s/n cut-off: 1.5; total intensity threshold: 0; minimum peak count: 1; precursor mass: 350–5000 Da. The peak lists were submitted to an in-house database search using Mascot 2.4 (Matrix Science), and were searched against the Uniprot-Swissprot database (May 30, 2015 update; 540,261 sequences; 191,876,607 residues) assuming 2 or less missed trypsin cut sites as well as fixed methylthio and variable oxidation and deamidation modifications. Target false discovery rate was set a 0.01. All identified proteins were assessed for gene ontology functions with an additional function set for structural components and those proteins with bone-related functions. Candidate proteins were selected if their relative abundances were greater than 1.5-fold (log_2_ scale) increased or decreased in cholesteatoma matrix compared to either middle ear mucosa or post-auricular skin for soft tissue analysis, or if proteins in cholesteatoma-involved bone were greater than 1.5-fold (log_2_ scale) increased or decreased relative to control ossicles and found to have ontologic functions corresponding to cellular metabolism, bone structure, bone metabolism, or epithelial content.

## Results

**Fig. 1:**
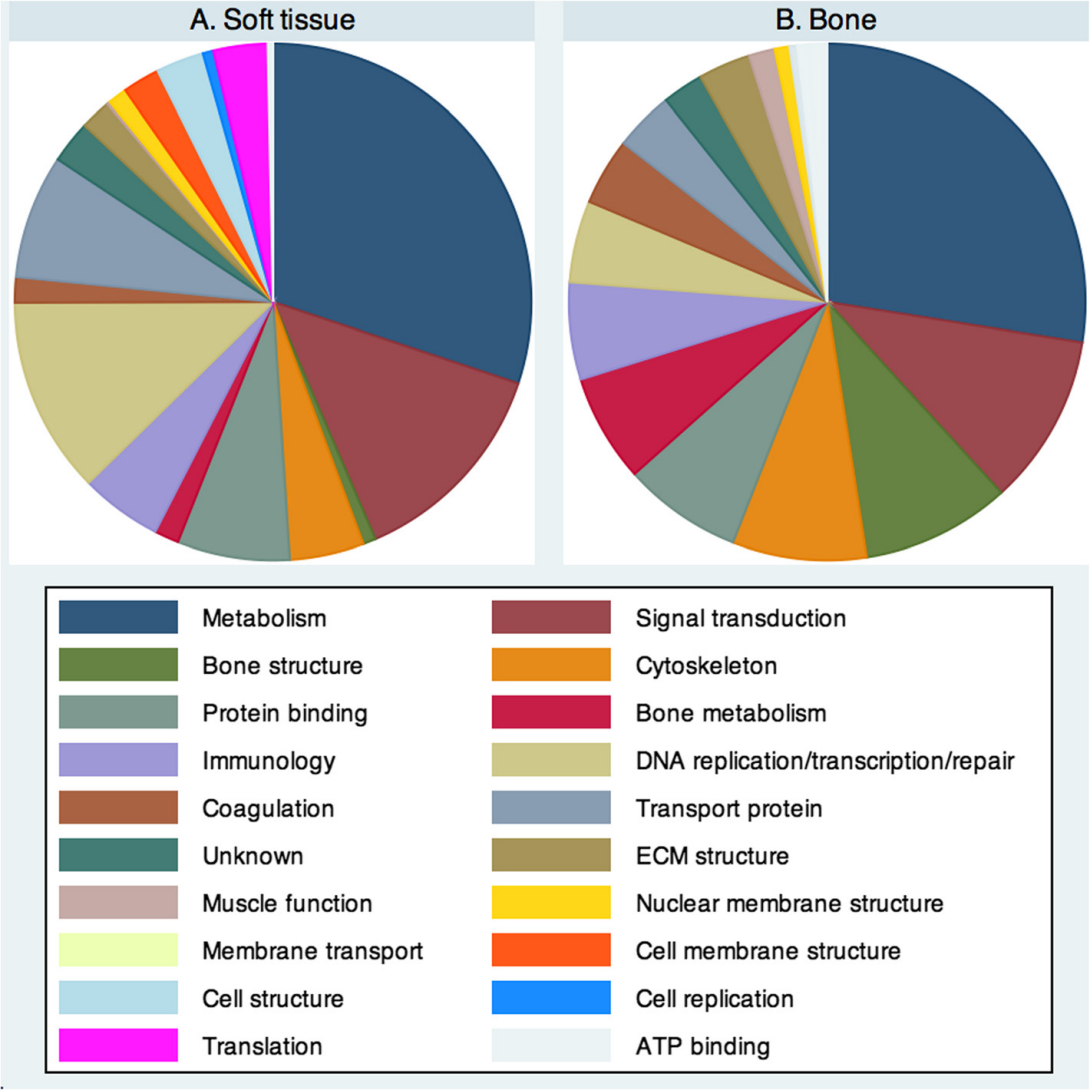
Ontologic functions of proteins identified in (**a**). Soft tissue analysis and (**b**). Bone analysis. General metabolic function and signal transduction properties represent the most common ontologic functions in both tissue sets, but wide variation exists among the remaining categories

The soft tissue arm of our study included representative samples from 12 cholesteatoma matrices, eight non-diseased middle ear mucosa fragments, and nine post-auricular skin samples. Healthy mucosa not involved with cholesteatoma could not be identified in four patients and was not collected; three post-auricular skin samples were suboptimal samples and not included. iTRAQ analysis of the included samples detected 10 720 spectra representing 8372 unique peptides. Setting stringency at 95 % probability of protein recognition and at least two peptides per protein identified 1490 proteins from 8315 of these peptides, corresponding to an average of 5.58 peptides per protein. The ontologic functions of these proteins are displayed in Fig. 1a. Cellular metabolism, signal transduction, and DNA replication/transcription/repair functions comprise more than 50 % of the identified proteins.

With respect to the bone lysate analysis, which included eight cholesteatoma-involved ossicles and eight control ossicles, 3159 spectra and 2392 unique peptides produced 480 proteins with at least two peptides and 95 % confidence of identification (4.96 peptides per protein). After subtracting protein clusters representing homologous protein family members, 428 were recognized with greater than 95 % confidence and further scrutinized. Gene ontology functions for the identified proteins are shown in Fig. 1b. The most common functions identified were metabolism, signal transduction, and bone structure, which accounted for 50 % of all proteins; 10 % of proteins played a role in bone structure and another 7 % in bone metabolism. In comparison to the soft tissue analysis there were 256 common proteins, indicating our study identified 1662 proteins from cholesteatoma and involved middle ear structures.

**Fig. 2:**
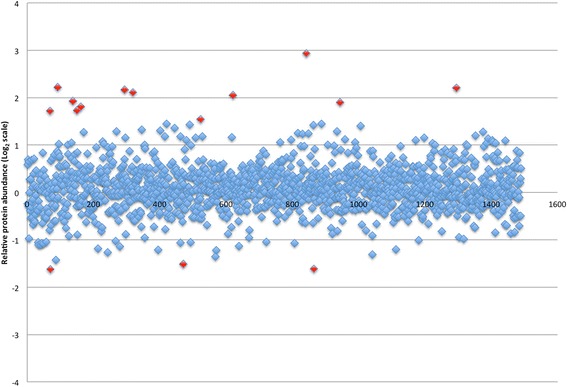
Logarithm (base 2) plot of relative abundances of all proteins identified in cholesteatoma matrix in reference to control mucosa. Positive values indicate greater protein content in cholesteatoma. The 15 proteins exceeding threshold abundances are indicated in red

**Fig. 3:**
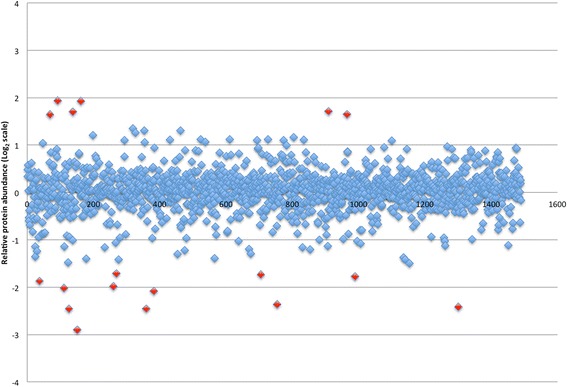
Logarithm (base 2) plot of relative abundances of all proteins identified in cholesteatoma matrix in reference to post-auricular skin. Positive values indicate greater protein content in cholesteatoma. The 18 proteins exceeding threshold abundances are indicated in red

**Fig. 4:**
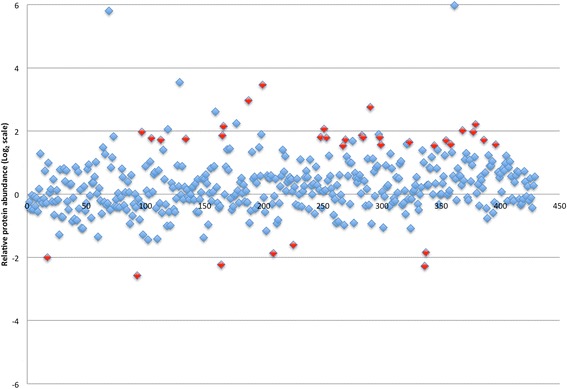
Logarithm (base 2) plot of relative abundances of all proteins identified in cholesteatoma-involved ossicles in reference to control ossicles. Positive values indicate greater protein content in cholesteatoma-involved bone. The 34 proteins exceeding threshold abundances and meeting inclusion criteria are highlighted in red

Figures 2 and 3 display the proteins identified in the soft tissue analysis with relative abundances of proteins in mucosa and skin, respectively, compared to cholesteatoma matrix. Proteins exceeding the relative abundance threshold are highlighted in red and represent potential candidates in cholesteatoma pathophysiology or biomarkers of disease. These 29 proteins are listed in Table 1 with their relative abundances in cholesteatoma matrix in reference to both uninvolved mucosa and post-auricular skin. Positive numbers indicate increased abundance in cholesteatoma while negative values indicate a higher relative abundance in normal tissue. Similarly, Fig. 4 shows all proteins identified in the bone analysis with potential candidates based on relative abundance alterations marked; Table 2 contains the 33 proteins marked in Fig. 4 sorted by relative abundance in cholesteatoma-involved ossicles to uninvolved ossicles. Four proteins were present in altered abundances in both the soft tissue and bone tissue analyses: creatine kinase B-type (CKB), tenascin-X (TNXB), serum amyloid P-component (APCS), and keratin type 2 cytoskeletal 8 (KRT8).

**Table 1:** Altered soft tissue protein abundances in cholesteatoma matrix relative to normal mucosa and post-auricular skin. Positive values indicate a greater abundance of a protein in cholesteatoma than the reference tissue. Italicized relative abundances indicate values below the detection threshold for one tissue but above detection threshold in the other. Study ID numbers correlate to Figs. 2 and 3

Protein name (study ID number)	NCBI gene ID	Number of unique peptides	Percent protein coverage (%)	Relative protein abundance	
				Mucosa	Skin
Bleomycin hydrolase (842)	BLMH	4	13.8	7.66	*1.14*
Protein S100-A9 (92)	S100A9	5	55.3	4.66	3.83
Acyl-coenzyme A thioesterase 1 (1295)	ACOT1	2	5.7	4.62	*−1.26*
Filaggrin (294)	FLG	10	3.7	4.50	*2.14*
FA binding protein, epidermal (319)	FABP5	7	65.2	4.32	*2.55*
Filaggrin-2 (621)	FLG2	3	1.0	4.15	*1.35*
Protein S100-A7 (138)	S100A7	7	54.5	3.81	3.25
Creatine kinase B-type (944)	CKB	2	7.9	3.74	*1.47*
Protein S100-A8 (162)	S100A8	5	64.5	3.50	3.80
Thymidine phosphorylase (150)	TYMP	10	35.1	3.32	*1.52*
Serine protease inhibitor B3 (69)	SERPINB3	22	50.3	3.30	3.12
Myelin protein P0 (523)	MPZ	6	25.0	2.92	*1.06*
Desmocollin-3 (989)	DSC3	3	4.2	*2.07*	−3.42
Ferritin light chain (965)	FTL	3	19.4	*1.42*	3.13
Fatty acid desaturase 2 (1301)	FADS2	2	3.6	*1.32*	−5.34
Hydroxymethylglutaryl-CoA synthase, cytoplasmic (705)	HMGCS1	2	6.0	*1.30*	−3.32
Fatty acid synthase (37)	FASN	38	23.7	*1.29*	−3.65
Collagen alpha-1(III) chain (754)	COL3A1	3	2.7	*1.26*	−5.15
Collagen alpha-6(VI) chain (260)	COL6A6	10	7.0	*1.18*	−3.95
Integrin alpha-M (909)	ITGAM	4	3.9	*1.16*	3.28
Keratin, type II cytoskeletal 79 (359)	KRT79	15	27.7	*−1.03*	−5.48
Tenascin-X (111)	TNXB	23	8.2	*−1.18*	−4.05
Serum amyloid P-component (269)	APCS	5	23.3	*−1.23*	−3.27
Tryptase alpha/beta-1 (382)	TPSAB1	4	14.5	*−1.65*	−4.23
Collagen alpha-2(I) chain (151)	COL1A2	7	54.5	*−1.86*	−7.48
Mimecan/osteoglycin (126)	OGN	7	24.8	*−1.89*	−5.49
Keratin type 2 cytoskeletal 8 (471)	KRT8	13	21.7	−2.85	*−1.55*
BPI fold containing family member B (865)	BPIFB1	3	8.5	−3.06	*1.68*
Fibrillin-1 (70)	FBN1	27	11.3	−3.08	*−1.23*

**Table 2:** Altered abundances of proteins found in the cholesteatoma matrix proteome relative to healthy ossicles. Positive values indicate increased protein abundance in cholesteatoma. Study ID numbers correlate to Fig. 4

Protein name (study ID number)	NCBI gene ID	Number of unique peptides	Percent protein coverage (%)	Relative protein abundance
				Normal ossicles
Isocitrate dehydrogenase 2 (199)	IDH2	3	4.6	11.02
Creatine kinase B type (187)	CKB	3	9.7	7.81
Thioredoxin domain-co8ntaining protein 5 (290)	TXNDC5	3	12.0	6.76
Cytoskeleton-associated protein 4 (379)	CKAP4	2	4.8	4.64
Protein disulfide-isomerase A6 (166)	PDIA6	5	15.2	4.45
Ribosome binding protein 1 (251)	RRBP1	3	2.4	4.19
Peptidyl-prolyl cis-trans isomerase (368)	FKBP10	2	4.5	4.06
ATP synthase subunit α, mitochondrial (97)	ATP5A1	9	21.2	3.92
Superoxide dismutase, mitochondrial (377)	SOD2	2	17.2	3.92
60S ribosome L14 (298)	RPL14	2	10.7	3.73
Stress-10 protein, mitochondrial (290)	HSPA9	3	12.0	3.73
Guanine nucleotide binding protein subunit β-2 (283)	GNB2L1	4	18.3	3.63
Malate dehydrogenase 2 (165)	MDH2	6	25.4	3.62
Serine protease inhibitor H1 (248)	SERPINH1	4	14.6	3.50
Dolichyl-diphosphooligosaccharide--protein glycosyltransferase (284)	DDOST	3	7.7	3.48
Elongation factor Tu (253)	TUFM	3	10.6	3.45
ATP synthase subunit β, mitochondrial (105)	ATP5B	6	14.6	3.41
ADP/ATP translocase 2 (134)	SLC25A5	8	24.8	3.37
Neutrophil elastase (269)	ELANE	2	6.7	3.31
Cathepsin G (113)	CTSG	5	22.4	3.29
Staphylococcal nuclease domain-containing protein 1 (386)	SND1	2	2.5	3.28
Very long chain specific acyl-CoA dehydrogenase (354)	ACADVL	2	3.5	3.26
Coatamer subunit gamma-1 (323)	COPG1	2	2.6	3.13
Prohibitin-2 (358)	PHB2	2	9.7	2.99
Lamin-B1 (396)	LMNB1	2	3.4	2.98
Valine—tRNA ligase (299)	VARS	2	2.1	2.97
Aspartate aminotransferase, mitochondrial (344)	GOT2	2	5.1	2.91
3-Hydroxyacyl-coA dehydrogenase type 2 (267)	HSD17B10	2	12.3	2.89
Membrane primary amine oxidase (225)	AOC3	4	3.7	−3.04
Keratin type II cytoskeletal 8 (337)	KRT8	3	6.2	−3.59
Collagen VIII, α-1 chain (208)	COL8A	3	5.0	−3.66
Cochlin (17)	COCH	11	20.4	−4.00
Keratin type II cytoskeletal 7 (164)	KRT7	6	13.4	−4.70
Tenascin-X (336)	TNXB	3	0.8	−4.84
Serum amyloid P-component (93)	APCS	5	21.5	−5.96

## Discussion

Our study used a mass spectrometry-based proteomic approach to evaluate the relative abundance changes between proteins found within middle ear mucosa and post-auricular skin relative to cholesteatoma matrix. A second arm of the study investigated the protein changes occurring in bone involved with cholesteatoma relative to healthy ossicles. We identified a number of potential candidates in the molecular changes among the functional components of the cholesteatoma matrix leading to uncontrolled growth and bony destruction.

Recognizing proteins with either increased or decreased abundance in both skin and mucosa reflect deviations from normal, particularly when they are functional rather than primarily structural in nature. Proteins fitting this description include members of the S100 family (A7, A8, and A9) and SERPINB3. S100 proteins are elevated in hyperproliferative skin disorders with primary roles in cell cycle regulation and cell differentiation [12, 13]. S100A7 also functions in an antibacterial capacity through the Toll-like receptor pathway in response to the bacterial protein flagellin [14], which is found in *P. aeruginosa* and *E. coli*, among other bacteria. SERPINB3 is a serine protease inhibitor associated with cellular atypia, evasion of apoptosis, and cholesteatoma proliferation [15–17]. Our identification of SERPINB3 and the three S100 proteins in cholesteatoma matrix agrees with existing literature noting increased levels in cholesteatoma relative to post-auricular skin [11, 18], but it is a potentially novel finding to find increased abundance relative to middle ear mucosa. Though adult cholesteatoma is a disease of keratinizing epithelium that likely originates from the lateral surface of the tympanic membrane or external auditory canal, its destructive properties occur primarily within the middle ear; as such, finding difference between the cholesteatoma matrix that allows it to overgrow mucosa and destroy neighbouring tissue may be more appreciable by analyzing the mucosa of presumably unaffected middle ear tissue.

A biomarker that is able to distinguish normal mucosa from cholesteatoma could prove useful for intra-operative tissue labeling or frozen section pathologic analysis, aiding the surgeon in removing all diseased tissue. Therefore another objective of this study was to recognize proteins that may function as indicators of residual or recurrent disease. As expected, the landscape of the cholesteatoma matrix proteome resembles that of normal skin, with multiple low-level abundance changes that likely reflect a complex interplay between various proteins and gene expression levels. The ideal candidate protein profile for this would have increased relative abundance in cholesteatoma compared to mucosa. Of the novel protein alterations we identified, some of the most intriguing changes involve BLMH, TYMP, FLBP5, and FLG/FLG2. BLMH represents a good candidate since it is located in superficial epidermis and maintains epithelial integrity [19]. Increased BLMH levels in cholesteatoma relative to mucosa, but unchanged in skin, is logical given that it is found primarily in the superficial and corneal layers of epidermis which are largely deficient in the non-keratinizing middle ear epithelium. This makes it an excellent potential biomarker since presence in the middle ear following cholesteatoma excision may represent residual disease. Likewise, FLG is long recognized as elevated in cholesteatoma by histopathologic studies relative to post-auricular skin [20, 21], and we note considerably greater degree of elevation in cholesteatoma compared to mucosa with similar change noted in FLG2, which is co-expressed with FLG during keratinocyte differentiation [22]. Previous data suggests an interaction between FLG and involucrin (IVL), a protein that creates a protective envelope around corneocytes, and increased IVL in cholesteatoma [21, 23]; we identified increased IVL in cholesteatoma matrix, but not at levels meeting detection threshold (1.97-fold increase).

TYMP abundance was elevated in cholesteatoma 3.25-fold in our study compared to mucosa, though less so compared to post-auricular skin. TYMP is an angiogenesis factor involved in avoiding hypoxia-induced apoptosis, though most research focuses on malignant disease [24]. The role of hypoxia in cholesteatoma is poorly understood and thought to stimulate matrix metalloprotease release and subsequent perimatrix degeneration [25]. Elevated TYMP may contribute to the independent growth capability of cholesteatoma particularly when oxygen requirements exceed that available from direct diffusion in aerated portions of the middle ear. In addition to oxygen supply, energy demand is another hurdle cholesteatoma must overcome to continue growth and local destructive potential. Fatty acid metabolism is gaining attention in cholesteatoma pathophysiology as an available energy source to propagate self-sufficient growth [25]. We found epidermal fatty acid binding protein (FABP5) is highly overabundant in cholesteatoma compared to normal mucosa (4.3-fold), with a lesser increase compared to skin. Initially identified in psoriasis, FABP5 is unique to keratinocytes, is a key factor in keratinocyte differentiation, and interacts with other proteins found at altered abundances in our study [26]. Of note, increased FABP5 stimulates increased IVL and is stabilized by S100A7 [27, 28]. FABP5 also appears to have a role in inflammatory change and tissue injury involving keratinocytes [29].

Other notable observations are the reduction of several structural proteins in cholesteatoma compared to post-auricular skin. These proteins provide insight into the molecular changes occurring that cause the well-recognized fragility of matrix intra-operatively. Desmocollin-3 (DSC3) likely contributes to this friability, as loss of function is found in skin fragility disorders [30], which matches the abundance profile we observed with decreased levels (3.42-fold) in cholesteatoma compared to skin, but above that seen in mucosa. Intercellular tight junction loss in cholesteatoma compared to normal skin has been shown by electrical impedence studies, agreeing with the impaired intercellular connections we found [31]. As shown in Table 1, a large proportion of the proteins reduced in cholesteatoma relative to skin are structural components.

With respect to the bone proteome of cholesteatoma-involved ossicles, the extensive number of changes mirrors the complexity of bone as an organ. Many of these changes relate to increased metabolic function, protein degradation, and inflammatory response and less so with structural components. A number of the proteins are constituents of the endoplasmic reticulum, which is abundant in osteoblasts for their synthetic function. Among the proteins with functional ontologic duties, Tenascin-X (TNXB), Serine protease inhibitor H1 (SERPINH1), and Neutrophil elastase (ELANE), are known factors in bone remodeling. TNXB, decreased in cholesteatoma-involved bone relative to normal ossicles and one of the proteins common to bone and soft tissue analyses, stabilizes extracellular matrix and collagen fibril formation; reduced TNXB is associated with abnormal collagen and elastin deposition and extracellular maturation [32–34]. Whether altered TNXB levels in our study reflect a pathologic or reactive change to cholesteatoma remains to be seen. SERPINH1 is a serine protease inhibitor molecular chaperone with critical function in endochondral bone and cartilage formation [35]. Elevated SERPINH1 in cholesteatoma-involved ossicles suggests either increased bone remodeling or repair of the affected bones. This is also likely the scenario for our observation of increased ELANE in cholesteatoma-involved bone. Involved in innate cellular immune processes, ELANE also functions in Collagen VI breakdown and matrix metalloprotease activation [36]. As noted in the soft tissue analysis, proteins present at increased abundances in cholesteatoma-involved bone may also represent markers of residual disease. Cytoskeleton-associated protein 4 (CKAP4) primarily functions in skin development and maintenance [37], implying its presence in cholesteatoma-involved bone correlates with presence of invasive disease.

Limitations of our study center on the use of a large-scale proteomic approach to the identification of these altered protein abundances. We found several abundance differences between cholesteatoma matrix and skin compared to the difference between matrix and mucosa. This is challenging to explain but may represent an alteration in protein composition in epidermis as cholesteatoma matures. One possible reason for this is that the local environment in the middle ear is different from that of the exposed post-auricular region. Alternatively, dermal elements could be underrepresented or absent in cholesteatoma while they would not be present in mucosa since there is no appreciable submucosa in the middle ear. Yet another consideration is the inability to measure proteins present in very low abundances, since the peptide fragments from these proteins fail to reach detection thresholds due to the presence of peptides from highly expressed proteins. This remains a challenge to all proteomic studies, although mass spectrometry-based techniques have a wider dynamic range than traditional 2DGE [10, 38].

Additional limitations specific to biomarker discovery studies warrant mention. Identification of disease biomarkers represents a chief objective of proteomics, though to date it as been difficult to carry putative biomarkers through to clinical practice, mainly due to variation between populations and study validity [39]. Discovery phase studies generally use samples containing well-defined and discrete disease states that do not necessarily reflect early, subclinical disease, which would be the ideal target for disease prevention. Similarly, patients with advanced disease often present in a condition that does not match the disease state used to identify the biomarker. Current candidates discovered with large scale proteomic approaches currently under inquiry for roles in clinical diagnostics and disease management include proteins altered in COPD [40], lung cancer [40, 41], gastrointestinal malignancy [42], transplant rejection [43], metabolic diseases [44]. Nonetheless, at present no single protein biomarker discovered through proteomics demonstrates sufficient accuracy to predict disease in a subclinical state [45]. Multiplex approaches to biomarker development present promise to increase understanding of diseases by simultaneously testing multiple proteins and observing abundance changes in concert, and to provide biomarker panels with increased accuracy [45].

## Conclusions

In conclusion, we used a large-scale, two armed proteomic approach to identify and quantify the changes in protein abundance levels in cholesteatoma matrix relative to normal middle ear mucosa and post-auricular skin, as well as in ossicles invaded by cholesteatoma compared to normal ossicles. Given the quantity of proteins involved in epithelial integrity and metabolism, our results provide several avenues for future research into cholesteatoma pathophysiology. Moreover, our inclusion of middle ear mucosa noted novel protein increases in matrix compared to post-auricular skin. One of the exciting findings in our study is the large number of potential biomarkers to use for evaluating residual or recurrent disease, in particular BLMH, TYMP, FLBP5, FLG/FLG2, and CKAP4. Our results describe several accessible proteins that could be used, if future validation demonstrates they are present in altered abundances in cholesteatoma compared to nascent mucosa, as potential therapeutic or diagnostic targets with the aim of reducing recurrent or residual disease and the need for second look tympanomastoidectomy. The greatest challenge with cholesteatoma surgery is eradicating disease without compromising function. Ideally, future cholesteatoma surgery could incorporate intraoperative or postoperative histopathologic margin evaluation using these biomarkers to reduce residual disease and determine timing of second look surgery.
